# Measuring Disability among Migrants with Washington Group Tools: Reflections for Field Use

**DOI:** 10.3390/healthcare10101860

**Published:** 2022-09-24

**Authors:** Marco Tofani, Giovanni Galeoto, Anna Berardi, Silvia Iorio, Antonella Conte, Giovanni Fabbrini, Donatella Valente, Maurizio Marceca

**Affiliations:** 1 Department of Human Neurosciences, Sapienza University of Rome, 00185 Rome, Italy; 2 Professional Development, Continuous Education and Research Service, Bambino Gesù Children’s Hospital, IRCCS, 00165 Rome, Italy; 3 Neuromed—Istituto Neurologico Mediterraneo, Istituto di Ricovero e Cura a Carattere Scientifico—IRCCS, 86077 Pozzilli, Italy; 4 Department of Medico-Surgical Sciences and Biotechnologies, Sapienza University of Rome, 00185 Rome, Italy; 5 Department of Public Health and Infectious Diseases, Sapienza University of Rome, 00185 Rome, Italy

**Keywords:** migrants, refugees, asylum seekers, disability, methodology, Washington group, functioning, activity, participation, mental health

## Abstract

Measuring disability among migrants is a significant challenge; however, there is no consensus on how to measure disability and functional limitations. The present study reports a methodological approach to measure disability in refugees and asylum seekers using Washington Group (WG) tools, namely the WG Short Set (WG-SS), the Short Set Enhanced (WG-SS-E), and the Extended Set on Functioning (WG-ES). We interviewed 161 migrants in different regions of Italy. The recommended threshold for each WG tool was used. We identified 13.7% of migrants with disabilities using the WG-SS, 21.7% using the WG-SS-E, and 31.6% using the WG-ES. Anxiety and depression were the main weights to identify migrants with disabilities (11.8%). The WG-SS does not measure mental health, and therefore we do not suggest its use in the field. However, the WG-SS-E, together with some questions on pain and fatigue, should be considered to identify migrants with a greater risk of disability.

## 1. Introduction

The World Report on Disability estimates that 15% of the world’s population lives with a disability [1]. According to the United Nations (UN) Convention on the Rights of Persons with Disabilities (UNCRPD) [2], people with disabilities include those who have long-term physical, mental, intellectual, or sensory impairments that may hinder their full, effective, and equal participation in society.

Migrants with disabilities represent an invisible group of individuals who are forced to leave their countries in particularly disadvantaged situations [3]. The lack of data and formal procedures to identify migrants with disabilities is recognized by the European Union (EU) and the UN High Commissioner for Refugees (UNHCR), with a negative impact on assistance, support, and healthcare service provision [4]. However, addressing the needs of persons with disabilities is fundamental to the achievement of the global sustainable development agenda [5], particularly sustainable development goal 3, “good health and well-being”, which focuses on developing good practices and guaranteeing good health and well-being for everyone. Data on migration and disability must be evenly structured since they serve to correctly inform the health policies of individual countries. In order to monitor progress on the 2030 Agenda, the international community unfortunately relies on disaggregated data on both disability and migration status. The inclusion of data on migrants with disabilities in statistics is crucial for the full and equal participation of this population in society. Being “visible” in statistics can enable inclusive disability policies and practices, as well as programs that result in more appropriate accommodations and better access to critical services, while also reducing marginalization and discrimination.

Limited evidence exists on the proportion of disability among migrant people, though it is acknowledged that migrant people have poor health outcomes, a greater risk of functioning and activity limitations, and restricted participation in society [6,7,8]. Refugees in particular have a significant risk of injury, abuse, and torture during the journey to the host country [9]. The most common diseases and issues are related to mental health, while one in six migrants experiences physical health problems [10]. At an international level, limited evidence exists regarding the prevalence of disability, with an estimated prevalence of 3–10% [11], while a recent study in Turkey revealed a higher prevalence of disability (24.7%) [12]. This variability may vary depending on the instruments used, context, and targeted population. In fact, the complexity of the concept has resulted in the proliferation of statistics on disability that are neither comparable nor easy to interpret. Furthermore, disability data are collected for different purposes, such as to estimate the prevalence of physical/mental impairments or to verify access to specific health or social services. Each purpose elicits a different statistic, and even when the intention is to measure the same concept, the actual questions used differs in ways that severely limit comparability.

At an international level, the most prevalent approach to estimate the proportion of people with disabilities is that proposed by the Washington Group (WG) on Disability Statistics [13]. The WG questions were designed to provide comparable data cross-nationally for populations living in a variety of cultures with varying economic resources. Domains were selected using the criteria of simplicity, brevity, universality, and comparability. It is expected that the information that results from the use of these questions will (a) represent the majority of, but not all, persons with limitation in basic actions; (b) represent the most commonly occurring limitations in basic actions; and (c) be able to capture persons with similar problems across countries [14].

The UN Statistical Commission and the UN Economic Commission for Europe’s Council of European Statistics have recommended the WG tool to collect disability information [15], and tools developed by the WG are now used in around 100 countries worldwide. However, the WG developed different tools for measuring people at a greater risk of disability, namely the WG Short Set (WG-SS) [16], the WG Short Set Enhanced (WG-SS-E) [17], and the WG Extended Set on Functioning (WG-ES) [18]. These tools are self-reporting measures that investigate the main aspects of functioning. The WG, together with the UN International Children’s Emergency Fund (UNICEF), has also developed two specific proxy measures to estimate the proportion of children aged 2–4 years and 5–17 years with disabilities. The Child Functioning Module (CFM) [19] is currently available in 12 languages and is used in different countries.

The WG tools are not medical tools; the focus is on measuring functioning in core domains, and it is in contrast to approaches that are based on impairments or loss in various body functions and structures, such as in the medical model of disability.

Considering the lack of consensus on measuring disability in migrant populations and the importance of estimating the proportion of migrants with disabilities, the present research aimed to compare WG tools and analyze differences between these tools in identifying migrant people with disabilities in the specific Italian context. We hypothesize that (1) the WG-SS may underestimate the population with disabilities because it does not take in consideration mental health–related issues, and (2) the WG-SS Enhanced and WG-ES can capture additional domains on functioning and allow identification of more functional impairments and persons with disabilities.

## 2. Materials and Methods

The present study was carried out by a research group at Sapienza University of Rome and the Rehabilitation & Outcome Measures Assessment (ROMA) association, in collaboration with the Italian Society of Migration Medicine. The research group has experience evaluating outcome measures and targeting specific interventions for those with disabilities and socially vulnerable groups [20,21,22,23,24,25].

### 2.1. Setting

Interviews were conducted in different reception centers in Italy involving different stakeholders. It is important to point out that the reception system has changed over time due to Italian government instability. In 2020, new legislation (Ref. [26]) set out that access to the Reception and Integration System (SAI–Sistema di Accoglienza e Integrazione) can be provided to refugees, asylum seekers, unaccompanied foreign minors, and foreigners entrusted to social services upon reaching majority age. Moreover, the SAI can also accommodate victims of disasters, migrants whose special civil value is recognized, holders of a residence permit for medical treatment, and holders of a special protection residence permit (recipients of social protection, victims of domestic violence, victims of labor exploitation). The primary objective of the SAI is to provide support for everyone in the reception system through individual programs designed to enable the individual to regain a sense of independence and thus enjoy effective involvement in life in Italy in terms of employment, housing, access to local services, and social interaction, as well as scholastic integration for minors.

### 2.2. Participants

A convenience sample of migrant persons hosted in different reception centers was selected in order to respect the following criteria: women and men, regardless of disability condition, aged 18 years or more, and having migrant status. Children and non-accompanied minors were excluded. The only exclusion criterion was the refusal to participate in the study. To recruit participants, the research team engaged different stakeholders working on the topic of migration. Objectives, procedures, and confidentiality issues were explained to potential participants, who had to give their consent to participate in the study.

### 2.3. Washington Group Tools

WG questions were designed to provide comparable data cross-nationally for populations of various cultures with varying economic resources. The questions reflect advances in the conceptualization of disability and the use of the International Classification of Functioning, Disability and Health (ICF) [27] as a conceptual framework. In a break from the biomedical approach to disability, the ICF presents a bio-psycho-social model that considers disability as the interaction between a person’s capabilities (functional limitations) and environmental barriers (physical, social, cultural, or legislative) that may limit their participation in society. WG tools use the ICF framework, focusing on activity limitations. Different tools have been developed over the last years to reflect the need and complexity of the purpose, target population, and context. Please see below for a more in-depth description of the tools used in the present study; questions and response patterns are reported in Table 1.

The *WG-SS* [16] is intended for use in censuses and surveys. It is composed of six questions, and the brevity of the module makes it useful for larger surveys and for disaggregating outcome indicators by disability status. A single question per functional domain is included, including difficulties in seeing, hearing, walking or climbing stairs, remembering or concentrating, self-care, and communication (expressive and receptive). The WG-SS can be used to gather information on the population aged 5 years and above, with a knowledgeable proxy respondent providing information for children. However, the tool was not specifically designed for use in children and does not include specific childhood issues; therefore, the CFM [19] should be used to study disabilities in children.

The *WG-SS-E* [17] obtains information on difficulties a person may have in undertaking basic functioning activities, including seeing, hearing, walking, or climbing stairs, remembering or concentrating, self-care, communication (expressive and receptive), upper body functioning, and affect (depression and anxiety). The WG-SS-E is comprised of 12 questions in the eight domains of functioning described above. The six WG-SS questions are included in the WG-SS-E.

The *WG-ES* [18] expands upon the WG-SS by asking about more functional domains and by asking more questions within each domain. The WG-ES includes questions in the following domains: vision, hearing, mobility, cognition, self-care, communication, affect (anxiety and depression), upper body function, pain, and fatigue. The WG-ES also includes additional questions in domains covered by the WG-SS-E, as well as questions on functioning with and without the use of devices/aids where applicable.

### 2.4. Administrative Procedures

To recruit participants, an initial email was sent explaining the objectives of the project to different stakeholders. Since SAI centers are directly appointed by a specific agency of the Italian government called Servizio Centrale (Central Service), an official communication was sent to request permission to proceed with the interviews. Once permission was obtained from the Central Service, migrants were interviewed. Interviews were conducted in person after three days of training on WG tools. Sociodemographic characteristics were collected during the interviews, together with legal status. Legal status was defined as follows: *asylum seeker*: someone whose request for sanctuary has yet to be processed; *refugee*: a third-country national who, owing to a well-founded fear of being persecuted for reasons of race, religion, nationality, political opinion, or membership in a particular social group, is outside the country of nationality and is unable or, owing to such fear, unwilling to avail himself or herself of the protection of that country, or a stateless person, who, being outside the country of former habitual residence for the same reasons as mentioned above, is unable or, owing to such fear, unwilling to return to it; *subsidiary protection*: an additional form of international protection that is complementary to refugee status and should only be granted if the requirements for refugee status are not satisfied [28]; *humanitarian protection*: a local type of protection granted in special and extraordinary cases where applicants are found not to be eligible for recognition as refugees or beneficiaries of subsidiary protection, but who are nonetheless considered to need protection due to special humanitarian reasons [29]; *undocumented migrant*: a third-country national present on the territory of a Schengen state who does not fulfil, or no longer fulfils, the conditions of entry as set out in Regulation (EU) 2016/399 (Schengen Borders Code) or other conditions for entry, stay, or residence in that EU member state [28]; or *special cases:* victims of trafficking, domestic violence, or work mistreatment [30].

### 2.5. Data Collection and Analysis

The three WG tools were used for capturing functional limitation in the same sample. Considering that WG tools have the same questions, we decided to use the WG-ES, which includes all questions of the WG, for interviewing migrant people, and then we selected specific questions to inform both the WG-SS and the WG-SS-E. Therefore, answers to both WG-SS and WG-SS-E derived from a secondary analysis of the WG-ES.

Before starting, the research group participated in an internal training course in order to level out confidence with the WG tools and to ensure consistency in administration and scoring. The first investigator (MT) led the training based on theoretical and practical activities. To measure how the training was effective, at the end of the training session, the research group participated in a practical test and discussed case studies.

As already mentioned, WG tools are used in more than 100 countries and are available in several languages. However, to minimize comprehension problems—even where respondents did not have a very good command of the available languages—the research team made use of language mediators when necessary. When necessary, language mediators were scheduled for a one-day course so they could be familiar with the research objectives and the questionnaires used. This made it possible to streamline the interview process and ensure a better approach to migrant people.

Sociodemographic characteristics were analyzed using frequency tables, mean, and standard deviation (SD). To measure disability, a standard threshold for each WG tool was used. For WG-SS, we considered the threshold to be ‘a lot of difficulty’ or ‘cannot do at all’ in any domain; for the WG-SS-E, we considered the threshold to be ‘a lot of difficulty’ or ‘cannot do at all’ in any domain and for upper body functioning, and ‘daily’ and ‘a lot’ in either domain for anxiety and depression. For the WG-ES, we also used the following thresholds: ‘every day’ and ‘a lot’ for pain, ‘most days’ and ‘all of the day’ for fatigue, or ‘every day’ and ‘most of the day’ or ‘every day’ and ‘all of the day’ for both. Cut-off thresholds are reported in Table 1. For data analysis, we used the recommended syntax of SPSS provided by the WG website for the WG-SS [31], WG-SS-E [32], and WG-ES [33]. Furthermore, we performed a prevalence ratio for gender. All analyses were then performed using Statistical Package for the Social Sciences (SPSS) version 20.0 (Chicago, IL, USA).

### 2.6. Ethical Considerations

Ethical approval was obtained from the Department of Human Neurosciences, Sapienza University of Rome. Informed written consent was sought from participants aged 18 years and above. Participants identified as having specific health needs, including rehabilitation and mental health services, were referred to local health authorities. Furthermore, those participants having a disability were provided information about the services available and how obtain access at the local level.

## 3. Results

The sample consisted of 161 people with a mean (SD) age of 29.8 (8.7) years. The majority (59.63%) were male and had a legal status of refugee or asylum seeker. Sociodemographic characteristics of participants are summarized in Table 2.

With respect to the proportion of migrants with disabilities, we found different values when using the three tools. Using the WG-SS, we identified 27 functional limitations in 22 migrants with disabilities; with the WG-SS-E, we found 51 functional limitations in 35 migrants with disabilities; and with the WG-ES, we identified 61 functional limitations in 51 migrants with disabilities. The major functional limitations were anxiety and depression (affect domain), as revealed using the WG-SS-E and WG-ES. Results are summarized in Table 3.

We also analyzed differences in the proportion of migrants with disabilities according to specific sociodemographic characteristics. Women had more functional limitations than men in each WG tool, with a prevalence ratio (PR) for disability of 2.62 using the WG-SS, 1.58 using the WG-SS-E and 1.86 using the WG-ES. Furthermore, disabilities were predominantly found in refugees and asylum seekers. Findings are summarized in Table 4.

## 4. Discussion

We reported one of the first investigations that analyzed the variability in measuring disability among migrants in Italy. We aimed to evaluate the differences in various WG tools for this target population. Our findings revealed that WG tools capture different functional limitations in people with disabilities, showing a ratio of 1.2:1 using both the WG-SS and the WG-ES, while the proportion was higher with the WG-SS-E (1.5:1). However, while the WG-SS-E can identify more functional limitations than the other WG tools, the WG-ES can identify more people with disabilities. The proportion of persons with disabilities identified was 13.7% (CI 95%: 8.7–19.9) with the WG-SS, 21.7% (CI 95% 15.6–28.9) with the WG-SS-E, and 31.6% (CI 95% 24.6–39.5) with the WG-ES.

These variabilities reflect theoretical constructs of the instruments because they analyze different domains of human functioning. Disability represents a complex process and is not a single static state. Developing statistics of disability is a challenge, and addressing all aspects related to disability, given the complex relationships among them and the varying social and cultural contexts that can affect how questions are interpreted, is a daunting task [34]. WG tools were designed to provide comparable data cross-nationally for populations living in a variety of cultures with varying economic resources [33]. However, questions should be tailored to capture differences according to specific needs and contexts. The choice of which questions to select should therefore reflect the research objectives and the target population.

Global refugee populations have been exposed to protracted psychological trauma, and the collective effect of these events on physical, emotional, and mental wellness is of great concern [35]. Kahn and colleagues reported that 16% of refugees present with musculoskeletal dysfunction alone, and over 60% live with mental health challenges [36]. Therefore, it is fundamental to use a tool that investigates mental health and psychosocial issues in this population. In fact, our analyses showed a higher percentage of disabilities in the affect domain (anxiety and depression) and confirms that mental health is a critical issue in migrants. This finding is in line with a study in Syrian refugees in Turkey, where a high prevalence of anxiety and depression was found, as may be expected in a conflict-affected, displaced population [12]. Therefore, the WG-SS could lead to a significant underestimation of the proportion of people with disabilities. In particular, mental health conditions are not only common but can also be more stigmatizing, resulting in greater barriers to participation or implications for wellbeing [37,38]. It is important to point out that WG tools are not designed for medical queries but were developed as set of questions on functioning for use in censuses and surveys. Functioning can be measured through different approaches and here were basically divided into self-reporting measures and clinician-based measurement or clinical assessment [39,40]. Self-reporting measures are questionnaire-based, low-cost, rapid to administer, and provide information on activity limitations and participation restriction, while clinician-based measurement is typically impairment-focused and often involves impairment assessment. They are time consuming, require trained clinicians and are expensive [41]. This paper explores the use of WG tools as a first-stage screening for disability in migrant populations. To identify specific health conditions, the use of clinical tools or evaluations is recommended.

With regard to methodological issues, a higher proportion of persons with disabilities were found across our analysis using the WG-SS-E and the WG-ES compared to the WG-SS. This finding is consistent with Mactaggart and colleagues [42], who analyzed the prevalence of disability in low- and middle-income countries and stated that this variability is to be expected considering the spectrum of functioning and functional limitations as described in the ICF, and due to the additional domains captured in the WG-SS-E and WG-ES. We found questions on anxiety and depression, together with those on pain and fatigue, useful to detect more people with functional limitations. In fact, there is evidence of a high prevalence of chronic pain or fatigue and their association with functional limitations and participation restrictions [43,44,45]. However, the WG-ES, which includes pain and fatigue, is probably too long to use in a census, though it can be used by non-governmental organizations as a special module for a more detailed analysis of disability [46]. Some authors recommend that alternate combinations of the domains of seeing, hearing, mobility, cognition, anxiety, and depression, plus pain and fatigue, should be tested to capture a greater proportion of people with functional limitations without substantially increasing the WG module length [43]. Different stakeholders should consider this aspect when working with migrants with disability and their specific context.

From a qualitative point of view, it is important to highlight that, within the same sample, the majority of people with disability were female: 63.6% in the WG-SS, 51.4% in the WG-SS-E, and 54.9% in the WG-ES. Furthermore, our data revealed that the proportion of people with disability using the WG-SS is 2.62-fold greater if a person is a woman, while the prevalence ratio was 1.58 and 1.86 using the WG-SS-E and WG-ES, respectively. Differences in the proportion of men and women with disabilities were less evident using the WG-SS-E and WG-ES, probably because these tools included mental health issues as well as pain and fatigue questions. Instead, when investigating only the main functional activities (seeing, hearing, mobility, cognition, self-care, and communication), women were more prone to showing a disability. This may be explained by the higher risk of physical and sexual violence in refugee women [47,48]. Furthermore, a large proportion of refugee women seeking gender-based violence response services had disabilities, and refugee women with disabilities are at a high risk of poor mental health [48]. More work needs to be done to determine how programs on physical and psychological health for violence survivors and protective services can become more inclusive and best meet the specialized needs of survivors with disabilities [49,50,51].

### Limitations

Despite these encouraging results, the present investigation has several limitations. First, we do not use a power analysis to determinate appropriate sampling; considering the explorative nature of the study, we opted to use a convenience sample. Furthermore, the relatively small sample size did not allow us to obtain robust evidence. A survey with a larger sample might help to better understand how WG tools work in different contexts. Second, we did not investigate disability in children and/or in non-accompanied minors, who represent a vulnerable population in need of specific physical and mental health services as well as inclusive education [52,53]. Another limitation is due to the fact that we did not investigate the possible relationship between migration route and disability. Although this was not the objective of our study, it could be useful to investigate whether there are any correlations between international travel security and the risk of functional limitations and disabilities. Further research should address these issues.

## 5. Conclusions

In conclusion, WG tools can capture different proportions of people with disabilities. The WG-SS may underestimate disabilities in migrants because it does not consider mental health issues. We suggest using the WG-SS-E or the WG-ES, depending on the objectives and specific context, since these tools provide a more comprehensive overview of disability. In some cases, it may also be useful to include pain and fatigue questions on the WG-SS-E to ensure no one is left behind.

## Figures and Tables

**Table 1:** Synthesis of the Washington Group tools.

WG Tools	Domains	N	Questions	Response Patterns	Cut-Off
**WG-SS**	Vision	1	Do you have difficulty in seeing, even if wearing glasses?	1. No difficulty 2. Some difficulty 3. A lot of difficulty 4. Cannot do at all	A lot of difficulty or cannot do at all in any domain
	Hearing	1	Do you have difficulty in hearing, even if using a hearing aid?		
	Mobility	1	Do you have difficulty walking or climbing steps?		
	Cognition	1	Do you have difficulty remembering or concentrating?		
	Self-care	1	Do you have difficulty with self-care, such as washing all over or dressing?		
	Communication	1	Using your usual language, do you have difficulty communicating, for example understanding or being understood?		
**WG-SS-E**	WG-SS	6	WG-SS	Pattern of WG-SS	A lot of difficulty or cannot do at all in any domain
	Upper body		Do you have difficulty raising a 2-liter bottle of water or soda from waist to eye level?	1. No difficulty 2. Some difficulty 3. A lot of difficulty 4. Cannot do at all	
		2	Do you have difficulty using your hands and fingers, such as picking up small objects, for example, a button or pencil, or opening or closing containers or bottles?		
	Affect (anxiety and depression)	4	How often do you feel worried, nervous, or anxious? How often do you feel depressed?	1. Daily 2. Weekly 3. Monthly 4. A few times a year 5. Never	Daily or a lot in either domain
			Thinking about the last time you felt worried, nervous, or anxious, how would you describe the level of these feelings? Thinking about the last time you felt depressed, how depressed did you feel?	1. A little 2. A lot 3. Somewhere in between a little and a lot	
**WG-ES**	WG-SS-E	12	WG-SS-E		
	Pain	2	In the past 3 months, how often did you have pain? Thinking about the last time you had pain, how much pain did you have?	1. Never 2. Some days 3. Most days 4. Every dayPattern Affect WG-SS-E	Pain: Every day and a lot Fatigue: Most days and all of the day or Every day and most of the day or Every day and all of the day
	Fatigue	3	In the past 3 months, how often did you feel very tired or exhausted? Thinking about the last time you felt very tired or exhausted, how long did it last? Thinking about the last time you felt this way, how would you describe the level of tiredness?	Pattern Pain WG-SS-EPattern Affect WG-SS-E	
	Others	20	Optional questions or questions related to assistive devices or medications		

WG: Washington Group; WG-SS: WG Short-Set; WG-SS-E: WG Short-Set Enhanced; WG-ES: WG Extended Set on Functioning.

**Table 2:** Sociodemographic characteristics of participants.

Age, Mean (SD)	29.8 (8.7)
**Gender**	**N (%)**
Male	96 (59.6)
Female	65 (40.4)
**Legal status**	**N (%)**
Asylum seeker	44 (27.3)
Refugee	45 (28)
Subsidiary protection	37 (23)
Humanitarian protection	14 (8.7)
Undocumented migrant	6 (3.7)
Special case	9 (5.6)
Prefer not to say	6 (3.7)

**Table 3:** Differences in the proportion of migrants with disabilities using WG tools.

	WG-SS		WG-SS-E		WG-ES	
Domain	N (%)	CI 95%	N (%)	CI 95%	N (%)	CI 95%
Vision	7 (4.3)	(1.7–8.7)	7 (4.3)	(1.7–8.7)	7 (4.3)	(1.7–8.7)
Hearing	1 (0.6)	(0.0–3.4)	1 (0.6)	(0.0–3.4)	3 (1.9)	(0.3–5.3)
Mobility	6 (3.7)	(1.3–7.9)	6 (3.7)	(1.3–7.9)	6 (3.7)	(1.3–7.9)
Communication	6 (3.7)	(1.3–7.9)	6 (3.7)	(1.3–7.9)	6 (3.7)	(1.3–7.9)
Self-care	2 (1.2)	(0.1–4.4)	2 (1.2)	(0.1–4.4)	2 (1.2)	(0.1–4.4)
Cognition	5 (3.1)	(1.0–7.1)	5 (3.1)	(1.0–7.1)	8 (4.9)	(2.1–9.5)
Upper body	/		5 (3.1)	(1.0–7.1)	5 (3.1)	(1.0–7.1)
Anxiety	/		14 (8.7)	(4.8–14.1)	14 (8.7)	(4.8–14.1)
Depression	/		5 (3.1)	(1.0–7.1)	5 (3.1)	(1.0–7.1)
Pain	/		/		3 (1.9)	(0.3–5.3)
Fatigue	/		/		2 (1.2)	(0.1–4.4)
Disability yes	22 (13.7)	(8.7–19.9)	35 (21.7)	(15.6–28.9)	51 (31.6)	(24.6–39.5)

**Table 4:** Differences in disability status according to sociodemographic characteristics.

	WG-SSDisability			WG-SS-EDisability			WG-ESDisability		
Gender	YES	NO	PR	YES	NO	PR	YES	NO	PR
Female	14 (8.7)	51 (31.7)	2.62	18 (11.2)	47 (29.2)	1.58	28 (17.3)	37 (23)	1.86
Male	8 (4.9)	88 (54.7)		17 (10.5)	79 (49.1)		23 (14.3)	73 (45.4)	
**Legal status**									
Asylum seeker	9 (5.6)	35 (21.7)		10 (6.2)	34 (21.1)		14 (8.7)	30 (18.6)	
Refugee	3 (1.9)	42 (26.1)		9 (5.6)	36 (22.4)		12 (7.4)	33 (20.5)	
Subsidiary protection	4 (2.4)	33 (20.5)		9 (5.6)	28 (17.3)		9 (5.6)	28 (17.3)	
Humanitarian protection	4 (2.4)	10 (6.2)		4 (2.4)	10 (6.2)		6 (3.7)	8 (4.9)	
Undocumented migrant	1 (0.6)	5 (3.1)		2 (1.2)	4 (2.4)		4 (2.4)	2 (1.2)	
Special case	1 (0.6)	8 (4.9)		1 (0.6)	8 (4.9)		2 (1.2)	7 (4.3)	
Prefer not to say	0	6 (3.7)		0	6 (3.7)		4 (2.4)	2 (1.2)	

## Data Availability

Not applicable.
